# Cell-Based Therapies for Cardiac and Vascular Regeneration in Cardiovascular Disease: Recent Advances, Translational Barriers, and Future Directions

**DOI:** 10.3390/biology15151260

**Published:** 2026-07-31

**Authors:** Sayan Paul, Raj Wasnik, Ranjith Kumavath, Tungki Pratama Umar

**Affiliations:** 1 Department of Microbiology and Immunology, University of Texas Medical Branch at Galveston, Galveston, TX 77555, USA; 2 Department of Biotechnology, School of Life Sciences, Pondicherry University, Puducherry 605014, India; rajwasnik00@gmail.com; 3 UCL Centre for Nanotechnology and Regenerative Medicine, Division of Surgery and Interventional Science, University College London, London WC1E 6BT, UK; tungkipratama@gmail.com

**Keywords:** cardiovascular disease, cell-based therapy, mesenchymal stromal cells, induced pluripotent stem cells, cardiac progenitor cells, exosomes, extracellular vesicles, paracrine signalling, tissue engineering, myocardial infarction

## Abstract

Cardiovascular diseases (CVDs) remain the foremost cause of death globally, responsible for 19.2 million deaths and 437 million disability-adjusted life years in 2023, with prevalent cases having more than doubled since 1990. No approved therapy restores myocardium lost to infarction. The adult heart replaces cardiomyocytes at approximately 1% per year in young adults, declining to about 0.45% per year with ageing, far below what is needed to recover the more than one billion cells destroyed by a large myocardial infarction. Cell-based regenerative strategies have been investigated for more than two decades, encompassing bone marrow mononuclear cells (BM-MNCs), mesenchymal stromal cells (MSCs), cardiac progenitor cells, cardiosphere-derived cells (CDCs), skeletal myoblasts, and induced pluripotent stem cell-derived cardiomyocytes (iPSC-CMs). Safety has been consistent. Efficacy has been modest and variable: the CADUCEUS trial demonstrated scar mass reduction with CDCs without proportionate ejection fraction improvement; the Phase 1/2 POSEIDON trial confirmed MSC safety in 30 patients; and the Phase 3 DREAM-HF trial, enrolling 537 patients, failed its primary endpoint (HR 1.2, *p* = 0.406). Mechanistic work has established that transplanted cells engraft poorly and exert their benefit principally through paracrine signalling mediated by secreted extracellular vesicles and exosomes carrying microRNAs, trophic factors, and immunomodulatory proteins. For iPSC-CMs, electrophysiological immaturity and arrhythmogenic risk in primate models remain unresolved barriers. Emerging strategies include CRISPR-engineered hypoimmune iPSC lines, bioengineered cardiac patches, injectable hydrogel scaffolds, and engineered exosome platforms. This review provides a comprehensive synthesis of preclinical and clinical evidence, examines translational barriers, and identifies the scientific and regulatory priorities required before these therapies can enter routine clinical practice.

## Simple Summary

Cardiovascular diseases are the leading cause of death worldwide, accounting for 19.2 million deaths in 2023. When a heart attack occurs, the affected cardiomyocytes die rapidly, and the adult heart replaces them at only about 1% per year, far too slowly to compensate for the loss following a large infarction. Current treatments stabilise patients but cannot rebuild lost muscle. Over the past two decades, clinical trials have tested stem cells from bone marrow, fat tissue, umbilical cord blood, and reprogrammed adult cells. These approaches are consistently safe. Some have produced modest improvements in cardiac function and scar reduction, but the transplanted cells rarely persist long enough to form new heart muscle. The benefit they confer appears to arise mainly from signalling particles they release called extracellular vesicles and exosomes, which carry microRNAs and proteins that reduce scarring, stimulate blood vessel growth, and dampen post-injury inflammation. This finding has opened a new research direction: engineering those particles directly, without transplanting cells at all. This review examines the full range of cell-based strategies studied to date, the clinical trial evidence, the barriers to progress, and what engineered vesicles, bioengineered tissue constructs, gene editing, and improved trial design might offer.

## 1. Introduction

Cardiovascular diseases (CVDs) remain the leading cause of death worldwide. According to the latest Global Burden of Disease report published in the *Journal of the American College of Cardiology* in September 2025, CVD itself caused 19.2 million deaths in 2023. That is one out of every three deaths worldwide. What is more, the total number of people living with CVD has more than doubled over the past three decades, rising from 311 million in 1990 to 626 million in 2023 [1]. Rising rates of obesity, type 2 diabetes and hypertension combined with ageing populations across every world region ensure that absolute burden will continue to grow even as age-standardised rates improve in higher-income lifestyles [1,2]. At the centre of this epidemiological problem is a biological limitation that remains unresolved despite decades of therapeutic effort: when cardiomyocytes are destroyed by myocardial infarction (MI), the adult human heart cannot replace them. Bergmann and colleagues established this with unusual precision in 2009 by using C^14^-isotope dating of human cardiac tissue to show that cardiomyocytes renew at approximately 1% per year at age 25, declining to approximately 0.45% per year by age 75, with fewer than 50% of cardiomyocytes exchanged over a normal lifespan [3]. A large infarction destroys over one billion cells within hours. Pharmacological therapy and percutaneous coronary intervention have meaningfully improved survival from acute events, but they address the obstructed vessel and not the downstream consequence of permanent contractile tissue loss that drives the transition to chronic heart failure [4,5].

What makes the problem solvable at least in theory is that cardiac regeneration is biologically achievable. The neonatal mouse heart regenerates completely following resection of up to 15% of the ventricular apex, which is driven by pre-existing cardiomyocyte proliferation and regulated by coordinated signalling among macrophages, sympathetic nerves and transcription factors including GATA4, Neuregulin1/ErbB4 and Tbx20 [6,7]. In mice, this regenerative capacity is largely lost by postnatal day 7 (P7), coinciding with cardiomyocyte binucleation and permanent withdrawal from the cell cycle [8]. The framing of “complete” neonatal regeneration should not be read as unconditional, however. Older comparative-embryology work indicates that the capacity for genuinely complete myocardial regeneration is lost earlier, and is narrower, than a blanket “through postnatal day 7” statement implies; in rabbits, the ability of the myocardium to regenerate fully after mechanical injury is lost by roughly day 24 of intrauterine development and by the first day of postnatal life, well before the classic P7 mouse cut-off usually cited in this literature [9]. Even within the canonical P1 mouse apical-resection model, the site of mechanical injury itself (e.g., the suture/ligation point) can retain persistent fibrosis, with true, scar-free regeneration confined to the distal ischaemic territory rather than the whole injured region (see Section 2.6). We therefore describe neonatal mammalian regeneration in this review as robust but incomplete and time-limited, rather than absolute.

In adult mice, genetic deletion of the Hippo-pathway component Salvador reverses post-infarction systolic failure; deletion of the hypoxia-responsive transcription factor Meis1 promotes cardiomyocyte cell-cycle re-entry; and suppression of adrenergic and thyroid-hormone signalling facilitates cardiomyocyte regeneration [8,10,11]. The biology is real and targetable. What has been missing is a method that is a safe, scalable, and clinically usable one that can switch on this dormant regenerative machinery in the injured human heart, and one that also addresses the vascular component of regeneration; restoring perfusion to viable but jeopardised myocardium is as central to functional recovery as replacing lost cardiomyocytes.

In the early 2000s, cell-based regenerative therapies emerged as the external approach to this problem. The hypothesis was simple: deliver living cells into the damaged myocardium and regeneration would follow. Early small-animal studies gave clues that bone marrow-derived cells could transdifferentiate into cardiomyocytes and generated immense fervour and launched a generation of clinical trials [12]. More than 200 trials and two decades later, the field has accumulated a safety record that is reassuringly consistent, a mechanistic understanding that has been substantially revised and an efficacy record that has forced genuine reckoning. The original hypothesis was wrong in a specific and instructive way because transplanted cells do not engraft in therapeutically relevant numbers, they do not differentiate into electromechanically coupled cardiomyocytes at scale, and the Lin-c-kit+ bone marrow cell transdifferentiation claims that launched much of the first wave of clinical work could not be reproduced by independent groups using rigorous lineage tracing [12,13]. The Phase 3 DREAM-HF trial is the largest conducted to date; it enrolled 537 patients and failed its primary endpoint (hazard ratio 1.17, 95% CI 0.81–1.69, *p* = 0.406 [14]. Meta-analyses of bone marrow mononuclear cell trials have found pooled ejection fraction improvements of 2 to 3 percentage points that have progressively shrunk as trial quality and blinding have improved, suggesting that earlier positive results were partly artefactual [15,16]. The c-kit cardiac progenitor cell programme, whose key publications were retracted following data integrity concerns, accelerated a sceptical regulatory environment that continues to affect the entire field.

Yet, to characterise two decades of cell therapy research as a failure would be to misread the evidence. Substantial evidence has established that several cell-based therapeutic platforms, including bone marrow mononuclear cells, mesenchymal stromal cells, cardiosphere-derived cells, and early induced pluripotent stem cell (iPSC)-derived cardiomyocyte preparations, have demonstrated favourable safety profiles in preclinical and early clinical studies, although their therapeutic efficacy continues to be actively investigated. The CADUCEUS trial demonstrated a sustained MRI-confirmed reduction in scar mass from 25.2% to 17.5% of left ventricular mass at 1 year following intracoronary administration of cardiosphere-derived cells. However, its allogeneic follow-on study, the ALLSTAR trial, was subsequently terminated for futility, highlighting the challenges in translating these promising findings into larger clinical studies [17]. The POSEIDON trial established that allogeneic mesenchymal stromal cells can be delivered without significant alloimmune response [18]. Perhaps most importantly, the mechanistic investigation triggered by the gap between poor cell engraftment and observed functional benefit has resolved into the paracrine paradigm: transplanted cells exert their effects primarily through secreted extracellular vesicles and exosomes carrying microRNAs, growth factors and immunomodulatory proteins that transiently reshape the post-infarction myocardial environment [12,19]. Cell-conditioned medium and isolated extracellular vesicles reproduce the functional benefits of cell transplantation in rodent models; chemically inactivated cells that maintain their structure but lose secretory capacity do not [19]. This is not a consolation explanation but a more traceable therapeutic basis than cell engraftment ever was because it shifts the design challenge from keeping cells alive in a hostile ischaemic environment to engineering defined molecular cargo in a manufacturable delivery vehicle.

That shift is redefining the field at precisely the moment this review is written. Several developments reported in 2024 and 2025 further extend the rapidly evolving landscape of cardiac regenerative therapy. Hu and colleagues demonstrated in *Nature Biotechnology* in 2024 that CRISPR-engineered hypoimmune iPSC-derived cardiomyocytes in which B2M and CIITA were disrupted and CD47 and PD-L1 were overexpressed survived long-term in fully immunocompetent allogeneic rhesus macaques without any immunosuppression [20]. Li and colleagues published in *Circulation* in 2024 the first demonstration of inhalable exosome therapy for cardiac repair [21]; Stem Cell-derived Exosome Nebulization Therapy (SCENT) delivers lung-spheroid-cell-derived exosomes via inhalation, exploiting the anatomical proximity of the pulmonary circulation to the heart to achieve cardiac-targeted delivery non-invasively, with efficacy shown in both mouse and swine MI models. A single-nucleus RNA-sequencing study identified a specific regenerative cardiomyocyte subpopulation in neonatal mouse hearts, designated CM4, characterised by active cell-cycle gene expression at 3 days post-MI and regulated by the transcription factors NFYa and SRF [22]; this remains directly relevant to iPSC-CM quality control. More recently, spatial transcriptomics has mapped the injury-dependent role of Lyz2-expressing endocardial cells, showing that sustained (rather than merely transient) Lyz2 activity in remote, non-infarct endocardial zones drives pathogenic lysosomal degradation of extracellular matrix and cardiomyocyte apoptosis in non-regenerative hearts, while genetic or pharmacological inhibition of this pathway preserves ECM and improves cardiac function after MI [23]. Deep learning architectures applied to spatial multi-omics datasets are generating predictive models of cardiomyocyte differentiation efficiency that begin to make patient-stratified cell therapy a realistic prospect [24,25]. Cardiac organoids now recapitulate MI hallmarks including cell death, fibrosis and impaired calcium handling under hypoxia–reperfusion protocols by providing a validated pre-clinical platform for testing cell-free therapeutic cargo [26]. Volumetric 3D bioprinting achieves Purkinje-like network architecture and heterogeneous cell density patterning approaching native cardiac structural complexity [27].

**Figure 1:**
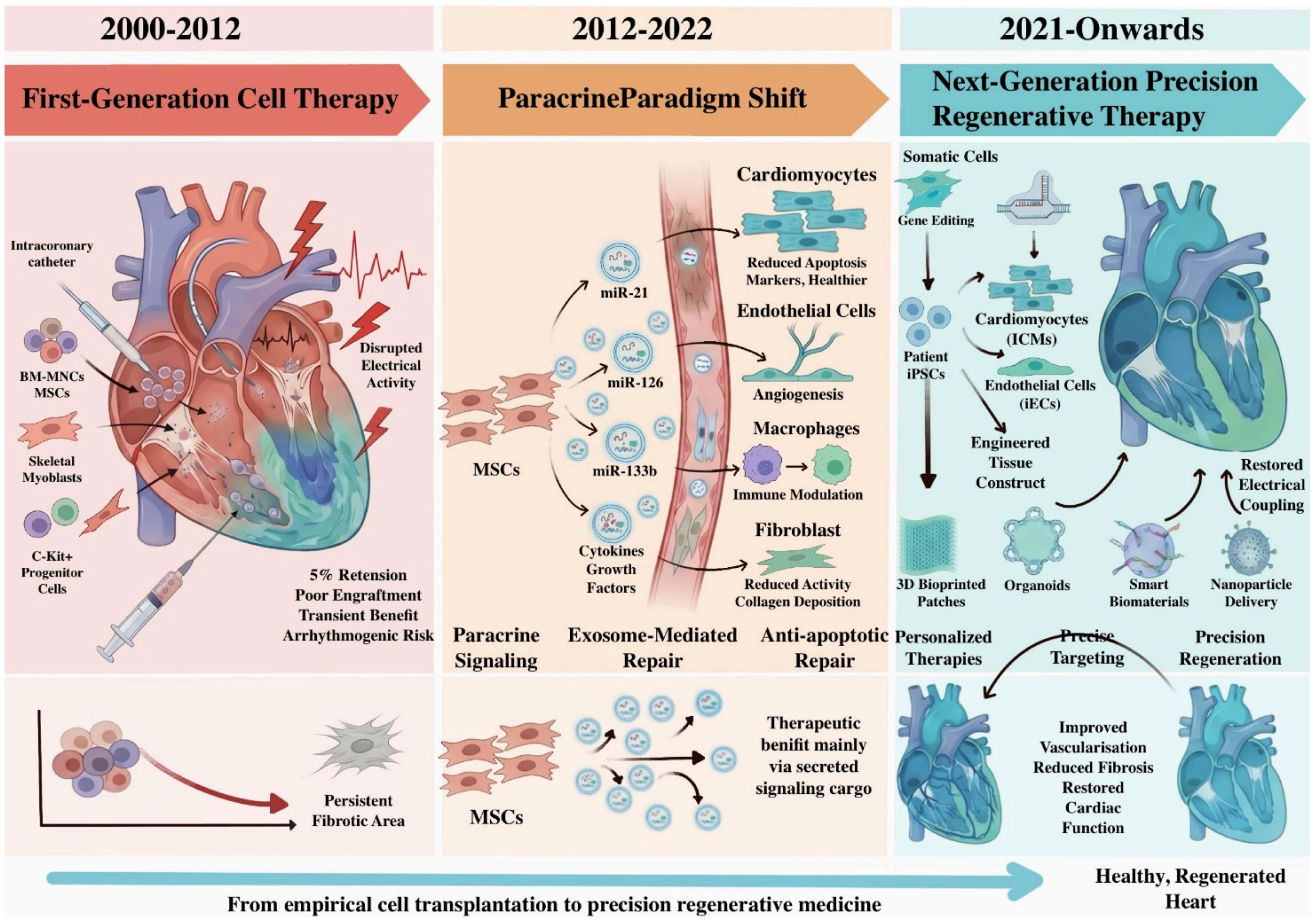
Evolution of cardiac regenerative therapy from direct cell replacement toward precision regenerative medicine. Early regenerative strategies assumed that transplanted cells would engraft and differentiate into cardiomyocytes, directly rebuilding lost myocardium. Clinical trials and mechanistic studies subsequently showed that long-term engraftment is minimal and that direct remuscularisation contributes little to observed functional benefit, prompting a shift toward the paracrine paradigm, in which the most therapeutic effect is attributed to secreted extracellular vesicles, exosomes, and other paracrine factors that reduce inflammation and fibrosis, promote angiogenesis, and support cardiomyocyte survival. Later-generation platforms such as hypoimmune iPSC-derived cardiomyocytes, engineered exosomes, inhalable delivery systems, spatial multi-omics, and AI-assisted patient stratification are designed to address the biological and translational barriers that limited first-generation cell therapies. [Figure illustrated using Procreate (Version 5.3.5)]. Created using Procreate (Savage Interactive Pty Ltd., Hobart, TAS, Australia; https://procreate.com; accessed on 8 June 2026).

None of these developments has been evaluated alongside the clinical trial record in a unified review. Most existing reviews either summarise what has been done in cell therapy without adequately addressing what has not worked and why, or address individual emerging technologies without placing them in the context of twenty years of clinical evidence. This review does both; it examines each cell type, delivery strategy and mechanistic concept with equal attention to what the evidence supports and where it fails or contradicts earlier assumptions. It gives particular weight to the unresolved contradictions that define the field’s current state: the DREAM-HF primary–secondary endpoint disconnection, the progressive deflation of BM-MNC efficacy estimation, the discrepancy between arrhythmias in primate iPSC-CM transplantation models and their apparent absence in early human case reports, and the specific implications of the allogeneic-outperforming-autologous MSC findings for manufacturing strategy. And it also evaluates the modern technological platforms like hypoimmune iPSC engineering, engineered and inhalable exosome delivery, single-cell and spatial omics, deep learning predictive modelling, cardiac organoids and advanced bioprinting as the scientific infrastructure of the next chapter of this field rather than as peripheral additions to an otherwise unchanged landscape. Finally, this review covers vascular regeneration alongside cardiomyocyte replacement wherever the mechanisms are shared or interdependent; paracrine angiogenic signalling (VEGF, HGF, SDF-1), co-transplantation of endothelial cells with iPSC-derived cardiomyocytes to support graft vascularisation, CD34+/CD133+ progenitor-cell trials in refractory angina, and CAR-T-cell approaches that reduce peri-infarct fibrosis and thereby improve vascular compliance are discussed in the relevant sections below. The conceptual evolution of the field, from early cell transplantation paradigms to precision regenerative medicine, is summarised in Figure 1.

## 2. Advances in Cell-Based Therapies for Cardiovascular Regeneration

### 2.1. Cell Types and Preclinical Evidence

#### 2.1.1. Skeletal Myoblasts (1990s–Early 2000s)

Skeletal myoblasts were the first cell type evaluated in cardiac cell therapy at clinical scale, given their ease of autologous isolation and ischaemia resistance. They do not express the cardiac gap junction protein Cx43 and therefore cannot integrate electrically with host cardiomyocytes. Clinical trials including the MAGIC trial were halted after documenting a significantly elevated incidence of ventricular arrhythmias in treated patients without durable functional benefit [28,29]. The lesson from skeletal myoblasts has been formative because the electrophysiological integration requirement for cells introduced into beating myocardium is not optional, and its absence produces harm. This lesson has directly shaped how rigorous arrhythmia safety testing requirements are for iPSC-CM programmes today. It is also why transient ventricular arrhythmias seen in primate studies of iPSC-CM transplantation are taken seriously and not dismissed as just a manageable side effect.

#### 2.1.2. Bone Marrow Mononuclear Cells and CD34+ Progenitors (Early 2000s)

Bone marrow mononuclear cells (BM-MNCs) were the second cell type tested in cardiac regeneration at scale, driven by early small-animal reports suggesting that Lin-c-kit+ sub-populations could transdifferentiate into cardiomyocytes after MI [12]. These reports could not be reproduced by independent groups using rigorous lineage-tracing methods, and the field eventually established that long-term cardiomyocyte transdifferentiation from BM-MNCs does not occur at any therapeutically relevant scale [12,13]. The functional benefits documented in animal models and some of clinical trials are now consistently attributed to paracrine effects like secreted VEGF, PDGF, IGF-1, extracellular vesicles and microRNAs that promote angiogenesis, reduce apoptosis, modulate the post-infarction inflammatory response and stimulate endogenous cardiac progenitor cells [12,15]. CD34+ and CD133+ haematopoietic progenitor cells have shown more consistent results in the specific indication of refractory angina, where intramyocardial delivery improved exercise tolerance and angina frequency in Phase 1/2 trials through direct endothelial differentiation and paracrine angiogenic signalling, demonstrating a genuinely vascular-regenerative application of cell therapy, distinct from cardiomyocyte replacement [30].

#### 2.1.3. Mesenchymal Stromal Cells (2000s–Present)

Mesenchymal stromal cells (MSCs) derived from bone marrow, adipose tissue, and umbilical cord are among the most extensively investigated cell products in cardiovascular regeneration. In experimental models of ischaemic injury, their benefits are mediated predominantly by paracrine signalling rather than durable cardiomyocyte replacement. Akt-modified MSCs and their conditioned medium protect ischaemic myocardium through secreted cytoprotective factors [31], while VEGF-expressing MSCs promote SDF-1-dependent recruitment of reparative cells and improve ventricular performance after infarction [32]. Isolated MSC-derived exosomes also reduce myocardial ischaemia/reperfusion injury, preserve cellular energetics, limit oxidative stress, and attenuate adverse remodelling [33,34].

Clinical studies indicate that both autologous and allogeneic MSC products can be delivered with acceptable short-term safety. In the POSEIDON trial, allogeneic MSCs did not induce clinically significant donor-specific alloimmune reactions, whereas the MSC-HF trial demonstrated reductions in left ventricular end-systolic volume and improvements in ejection fraction following autologous bone marrow-derived MSC treatment [18,35]. These findings support continued evaluation of standardised, off-the-shelf allogeneic products while recognizing that donor characteristics, manufacturing conditions, dose, and potency may influence therapeutic activity. Umbilical cord-derived MSCs remain attractive for scalable allogeneic manufacturing, although their comparative clinical superiority has not yet been established. The immune privilege of MSCs is also not absolute, and prolonged exposure or differentiation may alter immunogenicity. A separate trial of autologous adipose-derived stromal cells delivered by direct intramyocardial injection in patients with refractory angina (the MyStromalCell trial) reported sustained improvements in exercise capacity and angina symptoms at 3-year follow-up [36].

#### 2.1.4. Cardiac Progenitor Cells and Cardiosphere-Derived Cells (2000s–2010s)

Resident cardiac progenitor cells expressing the surface marker c-kit were identified in the early 2000s and attracted particular interest as endogenous precursors with potential for cardiomyocyte differentiation. The SCIPIO trial initially reported significant improvements in ejection fraction and scar size with autologous c-kit+ CPCs. These results were subsequently retracted following concerns about data integrity, and regulatory authorities discontinued several related programmes. The episode has had lasting consequences because it heightened scrutiny of cell identity and potency standards across the entire field and contributed to scepticism among both investigators and funders, which directly influenced the more demanding reproducibility requirements now embedded in FDA and EMA guidance.

Cardiosphere-derived cells (CDCs), generated by ex vivo expansion from endomyocardial biopsy specimens, represent a distinct and more potent programme. Preclinical dose-finding and post-conditioning studies in Yucatan and Sinclair mini-pigs established that intracoronary delivery of allogeneic CDCs (7.5–10 million cells) reduces infarct size and attenuates microvascular obstruction when given shortly after reperfusion in acute MI, directly informing the cell dose and delivery route later used in human trials [37]. A separate porcine study using intrapericardial administration of allogeneic CDCs (19 Large White pigs, 4 weeks post-infarction) showed reduced epicardial dense-scar expansion and improved electrical homogeneity at 16 weeks, providing large-animal proof-of-concept for a delivery route discussed further in Section 2.4 [38]. The CADUCEUS trial showed that delivering CDCs through the coronary artery reduced scar mass from 25.2% to 17.5% of left ventricular mass. At both 6 and 12 months, cardiac MRI also showed increases in viable myocardium and regional contractility. Global ejection fraction, however, improved only modestly, which is consistent with the mathematical relationship between scar size and ejection fraction rather than being evidence of direct remuscularisation [17]. The ALLSTAR trial (Makkar et al.) was a multicentre, randomised, double-blind, placebo-controlled Phase 1/2 trial of intracoronary allogeneic CDCs (CAP-1002) in 142 patients with prior MI, LVEF < 45%, and scar size ≥ 15% of LV mass. A pre-specified 6-month interim analysis showed a low probability of detecting a treatment effect on the primary endpoint (percentage change in infarct size by cardiac MRI), and the trial was stopped early for futility; the primary endpoint did not differ significantly between groups at either 6 months (*p* = 0.63) or 12 months (*p* = 0.39), although favourable secondary changes in LV remodelling parameters and segmental circumferential strain were observed [39]. Taken together, CADUCEUS and ALLSTAR illustrate a pattern that recurs across this field; a promising autologous Phase 1 signal (MRI-confirmed scar reduction) did not translate cleanly into a larger allogeneic efficacy trial, even though the underlying biology and safety profile were sound, a pattern that argues for choosing primary endpoints more carefully (se rather than for abandoning the cell type.

#### 2.1.5. Pluripotent Stem Cell-Derived Cardiomyocytes (2010s–Present)

Embryonic stem cells (ESCs) and induced pluripotent stem cells (iPSCs) are theoretically the most attractive sources for cardiac regeneration because of their capacity for unlimited self-renewal and their ability to differentiate into cardiomyocytes and other cardiac lineages at scale [40]. In large-animal and primate models, transplantation of iPSC-CMs or hESC-CMs has produced measurable remuscularisation with grafts, demonstrating progressive electromechanical coupling to host myocardium over months [41,42].

A recent non-human primate study reported graft stability at 12 weeks with evidence of structural integration and improved recovery from MI [40]. The Takahashi–Yamanaka reprogramming approach used Oct4, Sox2, Klf4 and c-Myc to convert adult somatic cells to pluripotency and made patient-specific autologous iPSC-CMs conceptually possible, which eliminated the embryo destruction requirement of ESC-based approaches [41].

In a porcine chronic-MI model, transplantation of hESC-CMs achieved substantial myocardial remuscularisation implants approximating a cubic centimetre in volume with stable engraftment, vascularisation, and minimal cellular rejection, but recipients also experienced frequent monomorphic ventricular tachycardia that resolved to normal sinus rhythm by 4 weeks post-transplantation; because the pig heart is roughly 7- to 8-fold larger than the non-human primate hearts used in earlier studies, arrhythmia burden was qualitatively worse than in smaller, faster-rate primate models [43]. Separately, a clinical-grade human iPSC-CM patch was evaluated in a porcine chronic-MI model by the Osaka group prior to first-in-human use, informing the manufacturing and delivery protocol subsequently used in the Japanese clinical case series discussed in Section 2.3 [44]. These porcine data sit between the non-human-primate and human evidence discussed below and support treating the primate arrhythmia signal as translationally relevant rather than a small-animal artefact.

The barriers to clinical translation of iPSC-CMs are specific and serious. The current differentiation protocols produce cells with a foetal rather than adult cardiomyocyte phenotype like immature ion channel expression, spontaneous automaticity, lower resting membrane potential, depressed inward rectifier current (IK1) and reliance on glycolysis rather than fatty acid oxidation [45,46]. These properties produce spontaneous ectopic firing and reduce the threshold for re-entrant arrhythmias when iPSC-CMs are grafted into beating adult myocardium. In multiple primate transplantation experiments, iPSC-CM grafts induced transient ventricular arrhythmias that resolved over weeks as grafts matured electrically [47]. This safety signal has not prevented early human clinical evaluation. Case reports from Japan have described functional improvement following epicardial iPSC-CM sheet implantation without documented arrhythmias [48]. In a landmark 2025 primate-and-human study, Jebran et al. demonstrated that epicardial engineered heart muscle (EHM) allografts constructed from iPSC-derived cardiomyocytes and stromal cells achieved long-term retention (up to 6 months) and dose-dependent structural and functional remuscularisation in rhesus macaques with and without MI-induced heart failure, without treatment-limiting arrhythmia or tumour formation, and that this same approach produced graft survival and structural remuscularisation in a first-in-human case with advanced heart failure [49]. The discrepancy between primate and human arrhythmia data is not explained and cannot be resolved without larger, systematically monitored human studies. A separate concern is tumorigenicity; residual undifferentiated PSCs or incompletely committed intermediates carry transforming potential that requires stringent quality control at the manufacturing stage [49]. Maturation strategies that address electrophysiological immaturity include 3D culture, electrical pacing, mechanical conditioning, metabolic switching from glucose to fatty acid oxidation, Brachyury transcription factor priming and cardiac fibroblast co-culture [45,46,47,50]. None has yet produced cells electrophysiologically indistinguishable from adult cardiomyocytes, and the minimum maturation threshold required for safe human transplantation has not been defined. These cell types, delivery routes, and key outcomes across the historical progression of clinical introduction are summarized in Table 1.

### 2.2. Mechanisms of Action: The Paracrine Paradigm

**Figure 2:**
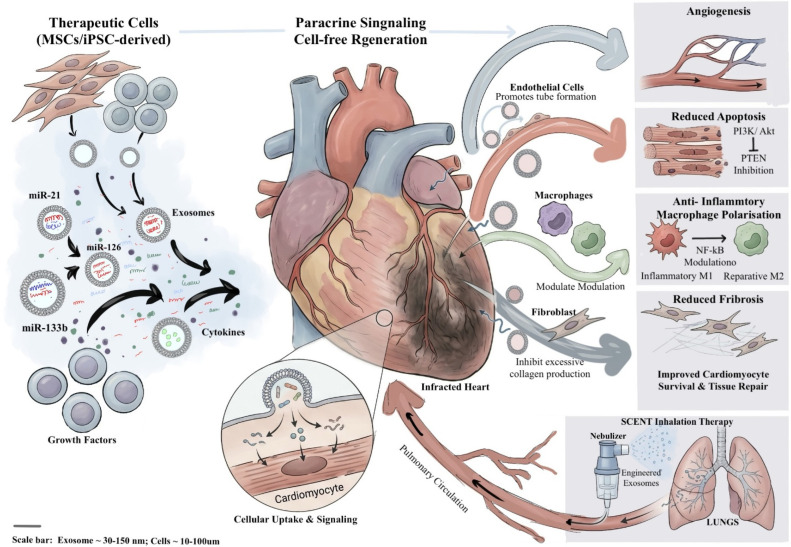
**Exosome-mediated mechanisms underlying cardiac repair following myocardial infarction.** Early regenerative strategies assumed that transplanted cells would engraft and differentiate into cardiomyocytes, directly rebuilding lost myocardium. Clinical trials and mechanistic studies subsequently showed that long-term engraftment is minimal and that direct remuscularisation contributes little to observed functional benefit, prompting a shift toward the paracrine paradigm, in which most therapeutic effect is attributed to secreted extracellular vesicles, exosomes, and other paracrine factors that reduce inflammation and fibrosis, promote angiogenesis, and support cardiomyocyte survival. Later-generation platforms such as hypoimmune iPSC-derived cardiomyocytes, engineered exosomes, inhalable delivery systems, spatial multi-omics, and AI-assisted patient stratification are designed to address the biological and translational barriers that limited first-generation cell therapies. [Figure illustrated using Procreate (Version 5.3.5)]. Created using Procreate (Savage Interactive Pty Ltd., Hobart, TAS, Australia; https://procreate.com; accessed on 8 June 2026).

The field’s most consequential mechanistic insight has been the resolution of an apparent paradox. Cell-tracking studies consistently show that fewer than 5% of injected cells remain in the myocardium at 24 h after delivery, and most are gone within days or weeks [12,13]. Despite this, functional improvements in ventricular function, scar size and perfusion have been documented in multiple animal models and some clinical trials. For years, the prevailing view was that the tracking methods were inadequate or that a small engrafted population had an outsized effect. The weight of experimental evidence now tells a different story; transplanted cells exert their benefit primarily while they survive, through the extracellular vesicles and exosomes they secrete, and the benefit ends when the cells do [12,19]. The major cellular targets and regenerative mechanisms associated with exosome-mediated cardiac repair are illustrated in Figure 2.

These extracellular vesicles, particularly exosomes 30 to 150 nm in diameter, carry a diverse cargo of microRNAs, messenger RNAs, proteins and lipids that are taken up by recipient cardiomyocytes, endothelial cells, fibroblasts and immune cells in the post-infarction milieu [12,19]. The key microRNAs implicated in the cardioprotective effects of MSC-derived exosomes include miR-21, which suppresses cardiomyocyte apoptosis through the PTEN/Akt signalling axis; miR-126, which promotes endothelial cell survival and neovascularisation (a directly vascular-regenerative mechanism); miR-133b, which inhibits fibroblast activation and collagen deposition; and let-7a, which modulates NF-kB inflammatory signalling [51,52]. MSC-derived extracellular vesicles also promote macrophage polarisation toward an anti-inflammatory M2 phenotype, reduce neutrophil infiltration in the infarct zone and inhibit T-cell activation by creating a local environment more permissive to endogenous repair [31,33].

The primacy of paracrine over structural mechanisms has been experimentally confirmed by showing that cell-conditioned medium and isolated extracellular vesicles reproduce the functional benefits of cell transplantation, whereas chemically inactivated cells with intact structural properties but absent secretory function do not [12,19]. This body of evidence has three direct consequences for the field that existing reviews have described individually but not followed through together. First, the relevant manufacturing quality attribute for an MSC product is not the cell number or viability but the potency of the secretome, a shift that requires entirely different batch-release assays. Second, if the benefit comes from what the cells secrete rather than from the cells themselves, then manufacturing the secreted cargo directly, without the cells, becomes the most efficient and controllable strategy. And third, the regulatory classification of a purified extracellular vesicle product is fundamentally different from that of a cell product, and this transition has not yet been resolved in any major regulatory jurisdiction (see Section 3.3). This mechanistic and practical comparison between cell-based and cell-free approaches is summarized in Table 2.

### 2.3. Clinical Trial Evidence

The clinical translation of cell-based cardiac therapies has generated more than 200 registered trials across cell types, delivery routes and patient populations. Safety has been consistently acceptable because the serious adverse events attributable to cell delivery are uncommon across MSCs, BM-MNCs, CDCs and CD34+ progenitors, and no cell type other than skeletal myoblasts has been associated with a consistently elevated arrhythmia risk in randomised trials [16,30,59].

BM-MNC trials were the first to generate large-scale clinical data. REPAIR-AMI, enrolling 204 patients with acute MI, reported a 2.5 percentage point improvement in ejection fraction at 4 months compared with placebo [16]. A separate, larger European Phase III trial, BAMI, randomised 375 patients with reperfused ST-elevation myocardial infarction to intracoronary autologous bone marrow mononuclear cell (BM-MNC) infusion or standard care. At 2 years, all-cause mortality did not differ significantly between the treatment and control groups (3.3% vs. 3.8%; *p* = 0.77), indicating that BM-MNC therapy did not confer a mortality benefit in this large randomised clinical trial [60]. When meta-analyses have examined the pooled BM-MNC literature, now comprising over 50 randomised trials, they have found average ejection fraction improvements of 2 to 3 percentage points, a magnitude that has progressively shrunk as trial quality has improved and that is now widely considered below the threshold of clinical meaningfulness [15,16].

MSC trials have produced a more consistent signal. The MSC-HF trial [35] in 60 patients with ischaemic heart failure demonstrated reductions in left ventricular end-systolic volume and improvements in ejection fraction at 12 months. POSEIDON, a Phase 1/2 study of 30 patients comparing autologous and allogeneic MSC delivery by transendocardial injection, confirmed safety with a 30-day serious adverse event rate of 6.7% and documented no significant alloimmune reactions at 1 year, an important finding given that most subsequent allogeneic MSC strategies depend on the absence of immune rejection demonstrated here [18]. DREAM-HF is the Phase 3 trial of mesenchymal precursor cells in 537 patients with heart failure with reduced ejection fraction, and it is the defining clinical result in the MSC field. It did not meet its primary endpoint of reducing recurrent non-fatal decompensated heart failure events [14]. Secondary analyses identified reductions in major adverse cardiovascular events (hazard ratio 1.17, 95% CI 0.81–1.69, *p* = 0.406) [14]. Secondary analyses identified reductions in major adverse cardiovascular events (MACE) in the NYHA class II subgroup, a finding that has been cited extensively and should be cited carefully; a subgroup effect identified post hoc, after a failed primary endpoint and with a point estimate running in the unfavourable direction on the primary analysis, requires a powered confirmatory study before it can support a therapeutic claim, and that study has not been done.

CardiAMP-HF is a Phase 3 trial (NCT02438306) testing potency-screened, high-dose autologous BM-MNCs delivered intramyocardially in patients with ischaemic HFrEF, selected using a validated cell-potency assay rather than an unselected cell dose [61]; a parallel allogeneic MSC arm targeting patients who lack favourable autologous cell characteristics began enrolling in 2023 but was paused that same year following an interim data and safety monitoring board review, and its outcome has not, to our knowledge, been fully reported at the time of writing [62]. This potency-guided selection strategy is conceptually important because it directly operationalises the paracrine-quality argument made in Section 2.1.3; rather than treating all autologous cells as equivalent, patients are screened for cells predicted to have adequate secretory function before treatment.

The CADUCEUS and its allogeneic follow-on ALLSTAR are discussed in detail in Section 2.1.4 above. iPSC-CM therapies entered early clinical evaluation primarily in Japan and more recently in the primate-and-human EHM programme from Göttingen [49]. A case report described improved cardiac function at 6 months following iPSC-derived cardiomyocyte sheet implantation without major complications [48], and the Jebran et al. *Nature* 2025 study reported graft survival and structural remuscularisation in a single first-in-human case as part of a combined primate-and-human dataset, not as a standalone human trial [49]. These remain single-patient or very small case reports; no powered randomised trial of iPSC-CM or EHM therapy yet exists, and the safety and efficacy profile in larger populations remains unknown.

Several consistent lessons emerge across all trial programmes. Patient-specific factors including age, diabetes and comorbidity significantly influence cell quality and therapeutic response, along with autologous cells from elderly or metabolically compromised patients often performing less well than allogeneic products from healthy young donors, a finding consistent with the paracrine secretome quality differences described above [51]. Cell delivery route matters because intramyocardial injection consistently achieves superior local retention compared with intracoronary infusion, though both result in the loss of most delivered cells within hours. The use of LVEF as the primary endpoint has consistently failed to detect a signal in trials that may have produced real biological effects, and the DREAM-HF experience illustrates how a trial can fail on its primary endpoint while showing secondary effects that, if confirmed, would be clinically important [14,63]. Representative landmark preclinical and clinical studies across cell types are summarized in Table 3.

### 2.4. Cell Delivery and Engraftment Strategies

Cell delivery route determines local retention, cell survival and the duration of paracrine signalling that drives therapeutic benefits. Intracoronary infusion is the least invasive approach and well suited to post-MI settings with patent vessels, but fewer than 5% of infused cells are typically retained in the target territory at 24 h due to washout during systole and mechanical loss. Intramyocardial injection, transendocardial via catheter or transepicardial during open surgery, achieves higher local concentrations and better retention, particularly in chronically scarred myocardium where intracoronary delivery would have no access [51]. Intravenous delivery distributes MSCs systemically and relies on homing signals to concentrate cells at the injury site; this route is convenient but results in the lowest myocardial concentrations of any approach.

Two further delivery routes are of specific clinical relevance. Retrograde coronary sinus (or coronary venous) delivery infuses cells or biologics backward through the cardiac venous system rather than the arterial coronary tree, exploiting the fact that total coronary-sinus occlusion for up to 60 min is tolerated because of Thebesian venous drainage. This route allows delivery independent of coronary anatomy or patency, a specific advantage when the infarct-related artery is chronically occluded and cannot be accessed and has progressed to clinical evaluation; percutaneous retrograde coronary sinus perfusion of autologous BM-MNCs in patients with chronic refractory angina was feasible and safe in a pilot study of 14 patients, with improvements in angina class and myocardial perfusion at 1–2 years [64], and the REVIVE trial subsequently tested retrograde bone-marrow-aspirate-concentrate delivery in ischaemic and non-ischaemic heart failure, finding LVEF improvements in all patients, but end-systolic diameter and BNP benefit were confined to the non-ischaemic subgroup [65]. Preclinical work indicates that retrograde delivery can achieve cell distribution across the left ventricle comparable to intramyocardial injection while allowing larger total cell numbers to be delivered safely [66].

Intrapericardial delivery administers cells or biologics into the pericardial space itself, a strategy of particular interest precisely because it does not depend on a patent coronary artery or vein and can therefore be used when the infarct-related artery is occluded and other routes are unavailable. A porcine study of intrapericardial allogeneic CDCs (19 Large White pigs, chronic post-infarction model) showed that this route hindered epicardial dense-scar expansion and promoted electrical homogeneity across the endocardial and epicardial scar at 16 weeks, providing large-animal support for a route that had previously been evaluated mainly in the context of arrhythmia substrate modification rather than as a primary regenerative-therapy delivery strategy [38]. Both routes remain earlier in clinical development than intracoronary or transendocardial delivery, but each addresses a specific access limitation for occluded or diseased coronary anatomy that the three conventional routes above cannot.

Biomaterial scaffolds represent the most significant engineering advance in improving retention and survival. Fibrin, hyaluronate, PLGA and synthetic hydrogels provide mechanical support, which mimic the extracellular matrix, protect cells from the hostile ischaemic and inflammatory environment and support sustained paracrine factor release [67,68]. Hyaluronate hydrogel co-delivered with hESC-derived cardiomyocytes in rat MI models demonstrated low arrhythmia rates, reduced ventricular remodelling and improved ejection fraction at 28 days [69]. Cardiac patch technologies combining iPSC-derived cardiomyocytes, cardiac fibroblasts and endothelial cells within biocompatible matrices have demonstrated improved electromechanical coupling and functional recovery in preclinical models compared with direct injection and electrical or mechanical preconditioning of patches before implantation, which further enhances cardiomyocyte maturation [30,50]. Preconditioning strategies that enhance cell survival and paracrine potency include hypoxic exposure, pharmacological conditioning with IGF-1, FGF-2 and TGF-beta1 and CXCR4 overexpression to improve myocardial homing [51].

The most consequential recent advance in delivery strategy is one that sidesteps the delivery problem entirely. SCENT, Stem Cell-derived Exosome Nebulization Therapy [21], published in *Circulation* in 2024, exploits the anatomical proximity of the pulmonary circulation to the heart because blood from the pulmonary vein flows directly to the cardiac chambers after gas exchange, making inhalation a biologically rational route for cardiac-targeted delivery [21]. Li and colleagues showed that lung-spheroid-cell-derived exosomes nebulised for inhalation improved cardiac function and reduced infarct size in mouse and swine MI models. Mechanistically, SCENT reduced CD36 expression in cardiac endothelial cells, identified by single-cell transcriptomics, altering lipid transport and inflammatory signalling in the ischaemic territory [21]. The approach is non-invasive, avoids the procedural risk and cost of catheterisation or surgery and critically enables repeated outpatient dosing that directly addresses the short in vivo half-life of exosomes that limits single-administration strategies. SCENT has already been discussed in recent reviews; here, it is considered specifically in relation to delivery route, repeat dosing, and the broader transition from live-cell products to cell-free cardiac therapeutics.

The broader shift from live-cell delivery toward acellular approaches extends beyond SCENT. MSC-derived extracellular vesicles and exosomes loaded with cardioprotective microRNAs reproduce or exceed the functional benefits of parental cell transplantation in multiple rodent MI models while avoiding immunological, arrhythmogenic and scalability challenges of live-cell products [51,52]. Bioengineered exosomes surface-modified with cardiac-targeting peptides or loaded with defined microRNA mimics have shown improved myocardial specificity and therapeutic efficacy in preclinical studies, pointing toward a generation of cell-free cardiac therapeutics defined by their molecular cargo rather than their cellular origin.

### 2.5. Gene Therapy and Direct Reprogramming

Gene therapy approaches to cardiac regeneration aim to modify cell behaviour by delivering therapeutic genes to the injured myocardium or by enhancing the regenerative properties of cells before transplantation. AAV vectors, particularly AAV1 and AAV9, have been used to deliver cardiac-specific transgenes, including SERCA2a, which restores calcium handling in failing cardiomyocytes; VEGF, which promotes therapeutic angiogenesis; and SDF-1, which enhances progenitor cell homing [70,71]. The Phase 2 AGENT-HF trial evaluating AAV1/SERCA2a showed a favourable safety profile and some evidence of functional benefit, though Phase 3 data have been mixed, and the field continues to work toward vectors with higher transduction efficiency and more durable expression [71]. Meta-analyses of intramyocardial gene therapy trials in chronic ischaemic heart disease show modest benefits that have not been replicated consistently across acute MI settings, and no Phase 3 trial has demonstrated definitive hard endpoint improvement [71].

CRISPR-Cas9 genome editing has added precision to both cell and gene therapy approaches. CRISPR activation (CRISPRa) has been used to transcriptionally upregulate endogenous cardiac transcription factors in fibroblasts by converting them into cardiovascular progenitor cells (ciCPCs) that can differentiate into cardiomyocytes in vivo, with transplantation of these cells substantially improving cardiac function and reducing scarring in mouse MI models [72]. Direct cardiac reprogramming through forced expression of the transcription factors Gata4, Mef2c and Tbx5 (GMT) converts postnatal cardiac fibroblasts into induced cardiomyocytes in vitro and in vivo [73,74]. The appeal of this approach is that it requires no cell transplantation. Instead, it redirects the large endogenous fibroblast population that accumulates in the post-infarction scar, steering it toward a potentially beneficial cardiac phenotype. Challenges include low reprogramming efficiency in human cells compared with mouse models, a discrepancy whose mechanism is not understood, and concerns about the immunogenicity and safety of viral vectors used for in vivo delivery. Synthetic mRNA-based strategies that transiently express cardiomyogenic transcription factors represent a potentially safer non-integrating variant [75].

### 2.6. Tissue Engineering, Cardiac Organoids, and Bioprinting

Tissue engineering seeks to overcome the fundamental limitations of single-cell injection by combining cells with biomaterials and bioactive molecules to create functional myocardial constructs [67,68]. Engineered heart tissue (EHT) constructs when seeded in fibrin or collagen hydrogels exhibit spontaneous beating, action potential propagation, and pharmacological responses that are consistent with native cardiac tissue [50]. Electrical field stimulation during 3D culture substantially improves the electrophysiological maturity of iPSC-CMs within these constructs, including ion channel expression, conduction velocity and gap junction coupling advancing the maturation challenge simultaneously with the structural engineering challenge [76]. Decellularised extracellular matrix (dECM) scaffolds derived from human or porcine heart tissue retain native myocardial architecture and provide a biologically relevant substrate for cell seeding, while 3D bioprinting with cardiac-specific bioinks allows spatially defined deposition of multiple cell types with architectural control at the micron scale [51,68].

While cardiac cell therapy has been widely studied, three developments from 2024 and 2025 have not been captured in any systematic evaluation, signalling a qualitative leap forward for tissue engineering technologies. First, hiPSC-derived multicellular heart organoids that under hypoxia–reperfusion protocols recapitulate key MI hallmarks, including cardiomyocyte death, fibrosis and impaired calcium handling, now serve as validated disease models for testing engineered exosome cargo and other cell-free therapeutics before animal studies [26]. These organoids bridge a historically costly gap between in vitro cell screening and in vivo animal experiments that has contributed to the poor translation rate of preclinical findings to clinical benefit. Second, AI-assisted 3D bioprinting incorporating machine learning for real-time defect detection during the printing process achieves an overall accuracy of 90.1%, enabling quality-controlled automated production of cardiac organoid constructs at a reproducibility level not previously possible with manual oversight [27]. Third, volumetric bioprinting techniques combining two-wavelength systems for alignment and curing achieve Purkinje-like network patterning and heterogeneous cell density distributions that approach the structural complexity of native cardiac conduction architecture, a level of spatial organisation not achievable with earlier extrusion-based or inkjet approaches [27]. A caveat follows directly from the discussion of incomplete neonatal regeneration in the Introduction. Even within the canonical P1 mouse apical-resection or coronary-ligation regeneration model, methodological work has shown that the site of mechanical injury (e.g., the suture or ligature itself) frequently retains localised fibrosis, while the ischaemic territory distal to it undergoes genuine functional and morphological regeneration, meaning that reported “complete” regeneration in these models can reflect the specific injury type and site examined rather than a universal absence of any scarring [77]. This distinction matters for tissue-engineering benchmarks; an organoid or EHM construct that recapitulates “complete” neonatal regeneration should specify which injury paradigm and which anatomical zone that claim refers to.

A separate and complementary contribution of this engineering period is the use of in vivo-produced chimeric antigen receptor (CAR) T cells targeting cardiac fibroblasts expressing fibroblast activation protein (FAP). Rurik and colleagues demonstrated in *Science* in 2022 that transient CAR-T-cell activity substantially reduced cardiac fibrosis and improved ventricular compliance in a mouse model of pressure-overload cardiomyopathy without adverse immune effects [78]. This approach does not replace lost cardiomyocytes but directly reverses the fibrotic remodelling that limits functional recovery following MI and cell therapy, improving vascular compliance as well as contractile mechanics, a further vascular-regenerative mechanism representing a complementary intervention that could enhance the effectiveness of concurrent regenerative cell therapies.

## 3. Challenges in Translating Cell-Based Therapies to the Clinic

**Figure 3:**
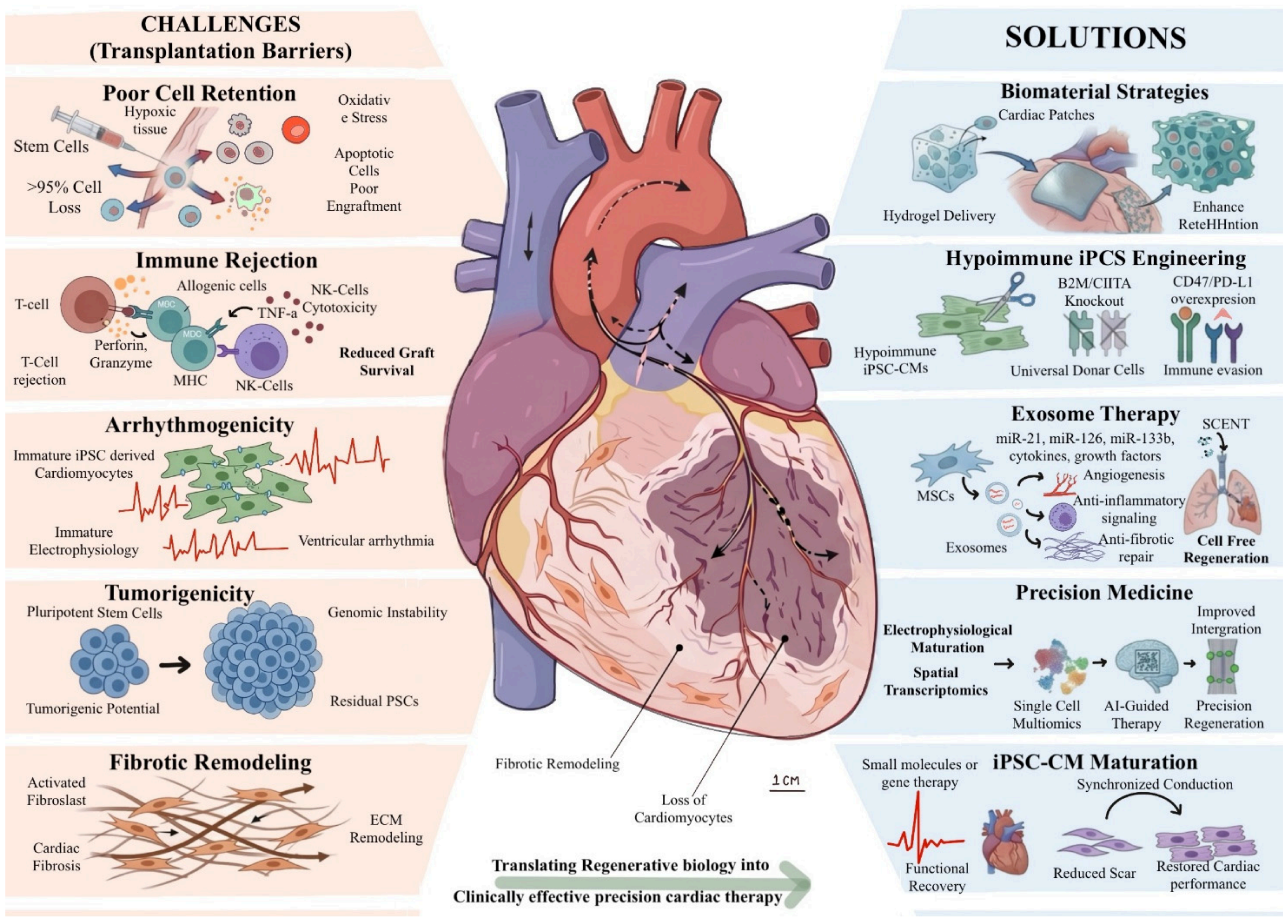
**Major barriers limiting successful clinical translation of cardiac regenerative therapies and emerging strategies designed to overcome them.** Early preclinical results were encouraging, but clinical translation has been limited by poor cell retention, immune rejection, arrhythmia risk, tumorigenicity, and ongoing fibrotic remodelling, individually and in combination, reducing graft persistence, integration, and durability of benefit. Candidate solutions under active investigation include biomaterial-based delivery systems, hypoimmune cell engineering, engineered exosome platforms, improved cardiomyocyte maturation protocols, and precision-medicine approaches informed by single-cell and spatial omics, with the shared goal of making cardiac regeneration safer, more reproducible, and capable of durable repair. [Figure illustrated using Procreate (Version 5.3.5)]. Created using Procreate (Savage Interactive Pty Ltd., Hobart, TAS, Australia; https://procreate.com; accessed on 8 June 2026).

The principal barriers limiting successful clinical translation and the emerging strategies developed to overcome them are summarized in Figure 3.

### 3.1. Immunological Barriers and Rejection

Immune rejection represents one of the most formidable barriers to cell-based cardiac regeneration, particularly for allogeneic and PSC-derived products. Allogeneic cell therapies trigger host immune surveillance via MHC mismatches, activating T-cell-mediated cytotoxic responses and reducing engraftment efficiency and graft survival [20,53,79]. MSCs occupy a special position because of their inherent immunomodulatory properties like low MHC class I expression, absence of class II and co-stimulatory molecules and constitutive secretion of immunosuppressive cytokines including IL-10, TGF-beta and PGE2, which all together suppress T-cell proliferation, inhibit NK cell cytotoxicity and promote regulatory T-cell expansion [31,79]. These properties have enabled allogeneic MSC delivery in multiple clinical trials without systematic immunosuppression. The important caveat is that MSC immune privilege is context-dependent; prolonged culture, in vivo differentiation, or exposure to high inflammatory cytokine concentrations can upregulate MHC class II expression, potentially converting a tolerogenic cell into an immunogenic one [80]. For patients with advanced chronic heart failure and where systemic inflammation is chronically elevated, the conditions for loss of MSC immune privilege may be more commonly met than in the acute MI settings studied in most trials.

For iPSC-CM transplantation the immunogenic challenge is qualitatively different and more severe. Undifferentiated or partially committed PSC derivatives express embryonic antigens absent from adult tissues and show aberrant MHC profiles that activate both adaptive and innate immune responses. In primate transplantation experiments, graft survival required effective immunosuppressive regimens, and lymphocytic infiltration was observed when suppression was withdrawn. The practical consequence is that iPSC-CM therapy in its standard form requires systemic immunosuppression in elderly patients with multiple comorbidities and substantially limits its applicability.

The hypoimmune iPSC engineering approach developed by Deuse, Hu, Schrepfer and colleagues addresses this problem at the molecular level. Simultaneous CRISPR-Cas9 disruption of B2M and CIITA—the beta-2-microglobulin gene required for MHC class I surface expression and the class II transactivator that regulates MHC class II expression— rather than the HLA genes themselves abolishes MHC class I and class II surface expression and eliminates the primary signal for T-cell-mediated rejection; combined, transgenic overexpression of CD47 signals to macrophages through SIRP-alpha as a “do-not-eat-me” signal, and PD-L1 inhibits T-cell activation through the PD-1 checkpoint pathway and generates cells that evade both adaptive and innate immune recognition [54,81]. In mice, hypoimmune iPSC-derived cardiovascular cell products survived long-term in fully immunocompetent allogeneic recipients without immunosuppression [54]. The 2024 *Nature Biotechnology* extension to fully immunocompetent allogeneic rhesus macaques demonstrated that hypoimmune iPSC-derived cardiomyocytes and endothelial cells survived at 4 months without immunosuppression and without tumour formation in a species whose own MHC system (Mamu), while genetically distinct from human HLA, is used as a stringent translational model precisely because rhesus macaques are outbred and immunologically intact, unlike most rodent models [20], providing the strongest current evidence that universal off-the-shelf allogeneic iPSC-CM products may be clinically achievable. What remains unknown is whether this tolerance is durable beyond 4 months in primates, whether it holds across all human NK-cell activation states and what the long-term safety profile of cells designed to avoid immune clearance will be in human recipients. These are not theoretical concerns. Exosome-based approaches sidestep the entire immune rejection question because exosomes lack surface MHC antigens and do not trigger cytotoxic immune responses at therapeutically relevant concentrations [82].

### 3.2. Technical and Manufacturing Limitations

The foremost biological challenge in cell therapy is poor post-transplantation cell survival, and the ischaemic myocardium is profoundly hostile because actively dying cardiomyocytes, intense neutrophil and macrophage infiltration, elevated oxidative stress, rapid ECM remodelling and oxygen deprivation combine to kill over 95% of injected cells within 24 h through apoptosis, necrosis and anoikis [79,83]. The window for therapeutic paracrine activity is consequently brief, and strategies that extend its preconditioning, scaffold delivery, and repeated dosing represent the most direct path to improving clinical outcomes without changing the fundamental cell biology.

Cell source quality introduces a second layer of variability that has significantly complicated clinical trial interpretation. Autologous cells from patients who are elderly, diabetic, or in heart failure are derived from the very conditions that impair cellular function because these cells show replicative senescence, reduced proliferative capacity, impaired paracrine activity and diminished differentiation potential [30,79]. Extending in vitro expansion to generate the cell numbers required for clinical administration only compounds these deficiencies, as epigenetic drift progressively alters gene expression over successive passages. This is the underlying biology that explains why allogeneic MSCs from young healthy donors can outperform autologous cells from the same patients they are being given to, a finding that should directly influence manufacturing strategy but has not yet driven a systematic shift in trial design [31,32].

For iPSC-CMs, the maturation problem is both a safety concern and a manufacturing challenge. Cells that are electrophysiologically immature carry arrhythmogenic risk; cells that have been successfully matured by electrical conditioning, mechanical strain and metabolic switching require longer and more expensive manufacturing processes, and the relationship between in vitro maturation metrics and in vivo arrhythmia risk in humans has not been validated [45,46,50]. Batch-to-batch variability in iPSC-CM differentiation efficiency and electrophysiological properties adds further complexity to dose selection and trial interpretation. Single-cell/nucleus RNA-sequencing approaches applied to iPSC-CM manufacturing lots are beginning to reveal the transcriptional heterogeneity within what are treated as uniform cell products, and comparison with the CM4 regenerative cardiomyocyte subpopulation identified in neonatal mouse hearts whose transcriptional signature marks genuinely proliferative cells could provide a quality metric for manufactured iPSC-CM products that is more biologically meaningful than current potency assays [22]. Similarly, deep learning models trained on spatial transcriptomics datasets from the cardiac injury border zone are beginning to predict differentiation efficiency and engraftment outcomes from pre-transplantation tissue profiles, pointing toward patient-stratified cell therapy in which the treatment is selected based on the recipient myocardial transcriptome rather than diagnosis alone [24,25].

From a manufacturing perspective, GMP-compliant production of sufficient cell numbers at reasonable cost remains technically demanding for all cell types. Cryopreservation introduces viability and function losses that have not been fully optimised. Automated bioreactor expansion can improve scalability but introduces its own sources of variability. For exosome products, the manufacturing challenges are different but not smaller; like isolation methods including ultracentrifugation, size-exclusion chromatography and tangential flow filtration, each have different yield-purity trade-offs, and there is no universally accepted GMP-compliant protocol for exosome manufacturing at a clinical scale [30,53]. These and other translational challenges are summarized in Table 4.

### 3.3. Ethical, Legal, and Regulatory Considerations

The ethical and regulatory landscape governing cell-based cardiac therapies has been shaped as much by the field’s internal failures as by external regulatory evolution. The c-kit cardiac stem cell controversy in which key publications reporting CPCs as a therapeutically important resident population were retracted following data integrity concerns had effects far beyond the specific programme it ended. It intensified regulatory demands for independent data verification, long-term follow-up requirements and stricter cell identity and potency characterisation standards that now affect every cardiac cell therapy programme regardless of its scientific relationship to the retracted work. The c-kit cardiac stem cell controversy illustrates how concerns regarding data integrity have strengthened regulatory expectations for independent validation, long-term follow-up, and rigorous characterisation of therapeutic cell products. These developments have contributed to more stringent standards governing the clinical translation of regenerative therapies.

The use of hESCs remains ethically contentious; their derivation requires the destruction of human embryos, raising bio-ethical and religious objections that have limited public funding in many jurisdictions and complicated international collaboration [57]. iPSCs circumvent this issue but introduce distinct concerns, particularly when CRISPR-Cas9 editing is employed; genomic instability, off-target editing events and for germline-capable pluripotent cells, the theoretical risk of heritable genetic modification, require stringent safety characterisation and informed consent processes that add time and cost to development [57,85]. The hypoimmune iPSC engineering approach multiplies these concerns by adding deliberate HLA disruption and checkpoint ligand overexpression to the standard iPSC safety profile, and the regulatory pathway for a product engineered to evade immune clearance in allogeneic recipients has not yet been defined [20,54].

Currently, no cell-based therapy has received FDA or EMA approval specifically for MI or heart failure. Most cardiac cell therapies remain at experimental or early clinical trial stages. Regulatory bodies including the FDA, EMA and ISSCR now mandate rigorous multicentre Phase 3 trials with GMP compliance, independent data monitoring and post-market surveillance before going to approval [57,58]. The absence of globally harmonised regulatory standards complicates cross-border trial approvals and adds redundant regulatory burden to programmes that seek simultaneous evaluation in multiple jurisdictions. The transition from cell products to exosome products introduces a classification question that regulators have not definitively resolved: are purified extracellular vesicle preparations biologics, drugs, or combination products? The answer determines the applicable manufacturing standards, preclinical safety package and clinical trial requirements, and the uncertainty is already impeding investment in exosome-based cardiac therapeutics [57,58].

### 3.4. Long-Term Efficacy and Safety

Questions about the durability of therapeutic benefit and the long-term safety of cell transplantation are central to whether any of these approaches will achieve regulatory approval. The accumulated evidence suggests that the modest functional improvements documented in many cell therapy trials are largely transient and consistent with temporary paracrine signalling from cells that do not engraft long-term [16,63]. In meta-analyses of BM-MNC trials, ejection fraction improvements observed at 3 to 6 months frequently diminish at 12 months and beyond. In DREAM-HF, the primary endpoint failure over a mean follow-up of 30 months is HR 1.17, *p* = 0.406, favouring neither arm on the point estimate, despite improvements in secondary endpoints at earlier time points, which fits the same pattern. This temporal trajectory should not be interpreted as evidence that cell therapy cannot produce durable benefit; it more likely reflects the fact that transient paracrine signals cannot permanently reverse a progressive fibrotic remodelling process without repeated dosing or a more durable therapeutic intervention.

Arrhythmogenicity is the most serious long-term safety concern, specifically for PSC-derived cardiomyocytes. The mechanism is well characterised; iPSC-CMs with immature electrophysiological profiles create ectopic foci through spontaneous automaticity and slow conduction in the grafted territory through poor gap junction coupling, predisposing to ventricular tachycardia or fibrillation [28,55]. Computational modelling by Yu and colleagues showed that arrhythmia risk is strongly influenced by the proportion of the infarct zone occupied by grafted cells and by their electrophysiological maturity, with large grafts of immature cells posing the highest risk [55]. In multiple non-human primate studies, transient ventricular arrhythmias following iPSC-CM transplantation have been documented and attributed to this mechanism [43,47,86]. These arrhythmias resolved over weeks as grafts matured, which is biologically plausible, but the window between transplantation and graft maturation represents a patient safety risk that has not been characterised in humans. The apparent absence of arrhythmias in the small number of human iPSC-CM case reports [48,49] could reflect genuine safety in human recipients, could reflect inadequate monitoring duration, or could reflect the small graft sizes used in these early cases. This discrepancy remains unresolved and it is not something we can ignore.

Tumorigenicity from residual undifferentiated PSCs or insertional mutagenesis from viral vectors used in reprogramming or gene delivery demands long-term surveillance in all treated patients [76,86]. Even autologous or hypoimmune cell therapy can provoke chronic inflammatory responses or adverse remodelling if graft–host coupling is incomplete or if the immune engineering strategy is imperfect. Beyond these cell-type-specific concerns, the inadequacy of left ventricular ejection fraction as a primary trial endpoint has become a structural problem for the field. LVEF can be influenced by how you image the heart, the patient’s loading conditions, and post-infarction remodelling trajectories, none of which have anything to do with whether a treatment actually works. It does not capture improvements in symptoms, hospitalisation, or cardiovascular death. The DREAM-HF experience, where a trial adequately powered for a clinical endpoint failed on that endpoint while showing secondary effects, may be real, and it is the clearest illustration of what happens when the wrong primary endpoint is chosen for a Phase 3 trial [14,63].

## 4. Future Directions

### 4.1. Engineered and Inhalable Exosome Platforms

The mechanistic clarification that paracrine signalling is the primary driver of benefit in cell-based cardiac therapy has opened a direct path toward cell-free therapeutic platforms that are, in several respects, more tractable than live-cell transplantation. Engineered exosome strategies aim to manufacture extracellular vesicles loaded with defined therapeutic cargo for direct myocardial delivery, eliminating the immunological, arrhythmogenic and scalability burdens of live-cell transplantation while preserving and potentially amplifying the regenerative signal [51,52]. The key microRNA targets like miR-21, miR-126, miR-133b and let-7a have been partially characterised, but whether optimal cardiac repair requires their co-delivery or whether a single dominant effector could be sufficient has not been determined. The effective human dose and dosing frequency for any engineered exosome product have not been established, and whether manufactured exosomes can replicate the full biological complexity of the hundreds of microRNAs, proteins and lipids present in native cell-secreted vesicles remains an open and important question.

Several engineering approaches are advancing in parallel. Electroporation enables loading of specific microRNA mimics or siRNAs at defined doses, providing standardised, quantifiable payloads. Surface modification with cardiac-targeting peptides, specifically peptides that bind to ischaemic cardiomyocyte surface proteins, improves myocardial biodistribution after systemic administration. Hybrid exosome–liposome constructs combine the biological membrane composition and cell surface molecules of natural exosomes with the drug-loading efficiency of synthetic lipid nanoparticles, potentially achieving both biological recognition and pharmaceutical-grade payload control [51]. Hydrogel-based local delivery systems that release exosomes at the infarct site over days to weeks address the short in vivo half-life of administered exosomes and provide sustained paracrine signalling without repeated interventional procedures [52].

SCENT (Section 2.4) remains the most conceptually novel delivery advance in this space. Whether SCENT will translate to human benefit is unknown, but the underlying principle leveraging existing anatomical delivery routes for cardiac-targeted, cell-free therapy has implications extending well beyond the specific SCENT protocol.

### 4.2. Hypoimmune and Universal Donor Cell Lines

The CRISPR-based hypoimmune iPSC engineering strategy developed over the past five years represents the most direct solution to the immune rejection barrier for allogeneic cell therapy and the 2024 *Nature Biotechnology* rhesus macaque data from Hu and colleagues makes it the strongest current candidate for achieving off-the-shelf allogeneic iPSC-CM therapy without immunosuppression [20]. The engineering approach of simultaneous B2M and CIITA knockout to eliminate MHC class I and class II expression combined with CD47 and PD-L1 overexpression to prevent NK-cell- and T-cell-mediated clearance produces cells that in mouse and primate models evade both branches of the adaptive immune response and the innate NK cell response [54,81]. Long-term graft survival in fully immunocompetent allogeneic macaques at 4 months without immunosuppression, without tumour formation and without inflammatory infiltration represents a genuinely encouraging safety and efficacy profile in a species used here as a stringent immunological model, not as a source of directly transferable data [20].

The unknowns are substantial and should be stated plainly; whether immune tolerance is maintained beyond 4 months in primates has not been reported. Whether CD47 overexpression provides durable NK cell evasion across all human NK cell activation states and subsets, including the activated NK cells present in the chronically inflamed milieu of heart failure, has not been established. The regulatory pathway for a product designed with engineered immune evasion has not been defined by any major regulatory authority, and the questions a regulator will ask are different from those of a standard allogeneic cell product, like how is long-term surveillance conducted for a cell that evades the immune clearance mechanism normally relied upon to limit aberrant cell survival? These are not arguments against developing hypoimmune iPSC-CM products, but they are the specific scientific and regulatory questions that the field needs to address to move this promising approach toward clinical trials responsibly.

### 4.3. iPSC-CM Maturation and Single-Cell Guided Quality Control

Resolving the electrophysiological immaturity of iPSC-CMs is a prerequisite for their safe cardiac transplantation, and the multi-factorial maturation strategies now in development represent a genuine convergence of bioengineering, cell biology and computational biology. Electrical field stimulation in 3D culture improves action potential upstroke velocity, conduction velocity and IK1 current density through mechano-electrical coupling that upregulates the adult ion channel programme [50]. Metabolic switching from glycolytic to oxidative metabolism, achieved by culturing iPSC-CMs in glucose-depleted fatty-acid-supplemented media, drives mitochondrial biogenesis, sarcomere alignment and electrophysiological maturation [45,46]. Brachyury transcription factor priming during early differentiation enhances structural maturation and T-tubule development [46]. Thyroid hormone supplementation and glucocorticoid treatment promote the developmental gene expression programmes associated with adult cardiomyocyte identity [45]. Combining electrical stimulation with mechanical cyclic strain has produced synergistic maturation effects exceeding either alone [50].

What remains absent from these maturation programmes is a validated quality metric that connects in vitro electrophysiological measurements to in vivo arrhythmia risk in humans. The CM4 cardiomyocyte subpopulation in neonatal regenerating mouse hearts characterised by high expression of immature markers TNNI-1, MYH-7 and ACTC-1 and strong activation of cell-cycle genes MKI67 and CCNB-1 at 3 days post-MI, with NFYa and SRF as upstream transcriptional regulators established by scATAC-seq [22], provides a transcriptional reference for genuinely proliferative cardiomyocytes at single-cell resolution. A follow-up study from the same group has since shown that NFYa also governs cardiomyocyte metabolism and proliferation during foetal heart development, extending this transcriptional framework from the neonatal to the foetal period [87]. Deep learning architectures applied to spatial multi-omics datasets are generating predictive models of cardiomyocyte differentiation efficiency [24,25]. Spatial transcriptomics has separately identified the role of injury-dependent Lyz2-expressing cells in remote, non-infarct endocardial zones [23], showing that sustained Lyz2 activity drives pathogenic ECM degradation and cardiomyocyte apoptosis in non-regenerative hearts. These findings overall offer a molecular roadmap for calibrating iPSC-CM maturation; manufactured cell lots could be quality-controlled against the transcriptional signatures of genuinely regenerative and genuinely mature cardiomyocyte states, and delivery timing could be optimised to coincide with endogenous repair programmes.

### 4.4. Combinatorial Approaches and Personalised Medicine

Single-modality cell therapy has not produced the clinical outcomes that the multifactorial pathobiology of post-infarction heart failure would demand of any single intervention. The disease involves not only the primary loss of cardiomyocytes but also microvascular dysfunction, pathological fibrotic remodelling driven by activated cardiac fibroblasts, neurohormonal dysregulation and a chronic inflammatory state that impairs endogenous repair. No single cell type or delivery strategy addresses all of these simultaneously [75,88]. Combination approaches that are scientifically motivated rather than empirically assembled represent the most plausible path toward durable clinical benefit.

Co-delivery of MSCs and iPSC-CMs combines the immunomodulatory, angiogenic and anti-fibrotic paracrine effects of MSCs with the direct contractile potential of maturing iPSC-CMs, and MSC co-transplantation may additionally reduce the arrhythmogenic risk of iPSC-CM grafts through local immunomodulation [75,88]. AAV-based gene therapy targeting SERCA2a or SDF-1 combined with cell transplantation addresses the calcium handling dysfunction in host cardiomyocytes that must electrically couple with transplanted grafts, potentially improving the integration environment [70,71]. Sequential delivery strategies that target the acute inflammatory phase with MSC-derived exosomes followed by the chronic remodelling phase with engineered anti-fibrotic agents address the temporal complexity of post-infarction biology. The CAR-T-cell approach targeting cardiac fibroblasts expressing FAP reduces the fibrotic substrate that limits functional recovery and could be combined with cell or exosome therapy to create a more permissive environment for regenerative activity [78]. Synthetic mRNA-based strategies that transiently upregulate cardiomyogenic transcription factors in endogenous cardiac fibroblasts, converting them in situ toward a cardiomyocyte-like fate, represent a combination-independent approach to regeneration that avoids cell transplantation entirely [78].

Personalised medicine frameworks are beginning to move from aspiration to specific scientific proposals. Genomic profiling of the cardiac substrate using single-cell and spatial transcriptomics before intervention could identify patients with residual viable myocardium in border zones that would benefit most from cell-based approaches, while identifying patients with predominantly acellular fibrotic scarring who would benefit less and who might respond better to anti-fibrotic or angiogenic strategies [22,23]. Computational arrhythmia risk models built from pre-transplantation cardiac imaging and transcriptomics data could predict individual patient risk from iPSC-CM grafts and inform dosing and maturation requirements before transplantation [55,56].

## 5. Landscape of Ongoing Clinical Trials

Trial registries are dynamic, and status can change between the time this section is written and the time it is read; the summary below reflects the most recent status information available to us and should be verified directly against ClinicalTrials.gov (or the relevant national registry) before being relied upon for citation in downstream work. Selected ongoing and recently paused trials are listed in Table 5.

No exosome or SCENT-type inhalable platform had, to our knowledge, entered a registered human cardiac trial at the time of writing; the ongoing-trial landscape for cell-free platforms therefore currently lags several years behind that for live-cell products, despite the mechanistic case (Section 2.2, Table 2) for eventually favouring cell-free manufacturing. This gap is itself informative; it indicates that regulatory and manufacturing standardisation (Section 3.3, Table 2) is currently the rate-limiting step for exosome translation, not remaining biological uncertainty.

## 6. Limitations of This Review

This is a narrative, not a systematic, review, and its literature selection carries the subjectivity inherent to that design. We searched major databases (PubMed, Web of Science, and trial registries) for English-language publications through early 2026, supplemented by citation tracking from key papers; we did not apply a formal systematic-review protocol (e.g., PRISMA), pre-register a search strategy, or use dual independent screening, and a differently constituted author team searching the same period could reasonably include or weight some studies differently. Second, given the size of the reference list, readers using this review to source primary literature are encouraged to verify each citation against the original publication before relying on it. Third, this review relies more heavily on secondary literature (other reviews and commentaries) for background and synthesis statements than would be ideal, particularly for very recent (2024–2025) developments where primary trial data are still emerging; we have prioritised primary sources wherever they exist and were available to us, but for some claims, particularly around ongoing trials (§5) and not-yet-published mechanistic extensions, secondary sources were the only available option at the time of writing.

Fourth, publication and reporting bias likely affects the underlying evidence base itself, independent of how we selected sources; negative or null preclinical results are known to be underreported in this field, and this may cause the preclinical literature we cite to overstate average effect sizes relative to what would be observed with complete reporting.

Finally, the ongoing-trials landscape (§5) is, by construction, a snapshot; trial status, enrolment, and results change continuously, and the specific trials listed there should be treated as illustrative examples of current activity rather than a complete or current registry extract.

## 7. Conclusions

Two decades of clinical investigation into cell-based cardiac regeneration have produced a scientific record that is more nuanced and more instructive than either its enthusiasts or its critics have tended to acknowledge. The field has not delivered the clinical breakthroughs that early results promised. Two decades of clinical investigation into cell-based cardiac regeneration have produced a scientific record that is more nuanced and more instructive than either its enthusiasts or its critics initially anticipated. The DREAM-HF Phase III trial, the SCIPIO retraction, the ALLSTAR futility analysis, and the progressive reduction in estimated BM-MNC efficacy as trial quality improved underscore both the biological complexity of cardiac regeneration and the importance of rigorous experimental and clinical study design. But the field has produced something equally valuable: a mechanistic understanding of cardiac repair that was not available before the cell therapy era, and a set of therapeutic tools whose scientific basis is substantially more rigorous than what preceded them.

The most important of these understandings is the paracrine paradigm. Transplanted cells do not rebuild myocardium by becoming cardiomyocytes; they create a transient regenerative environment by secreting extracellular vesicles and exosomes whose molecular cargo reduces apoptosis, attenuates fibrosis, promotes angiogenesis and vascular repair, and modulates the post-infarction inflammatory response [12,19]. This mechanism explains both what has worked in cell therapy, temporary functional improvements correlated with secretome activity, and what has not, durable structural regeneration that requires long-term cardiomyocyte replacement. It directly implies that the most tractable therapeutic strategy is not improving cell engraftment but engineering the exosomal cargo itself, a transition that is already well underway and that is represented in this review by engineered exosome platforms, inhalable SCENT delivery, and the hydrogel-based sustained-release systems (Table 2, Section 2.2 for a direct comparison against cell-based products, and Section 5 for the current, still cell-dominated, state of clinical translation) [21,51].

Three things remain genuinely unresolved and define the boundaries of what the field currently knows. First, the minimum iPSC-CM electrophysiological maturation threshold required for safe human transplantation has not been defined. The arrhythmia data from primate models are real, the single-cell transcriptomics tools to characterise maturation are now available, and the computational arrhythmia risk models to translate maturation metrics to clinical safety predictions are in development [22,43,45,46,55,56]. Second, the DREAM-HF NYHA Class II secondary finding requires a powered confirmatory trial and, given that the trial’s primary point estimate (HR 1.17) numerically favoured the sham arm, this confirmatory requirement should be treated as a precondition for clinical use of the NYHA II subgroup finding, not an optional next step [14]. Third, the regulatory classification of engineered exosome acellular products, manufactured from cellular processes, carrying defined molecular cargo, has not been determined in any major jurisdiction, and this uncertainty is actively impeding the investment and development work that the strongest scientific signal in the field now warrants [57,58].

The science of cardiac and vascular regeneration has advanced further in the past five years than in any comparable previous period. Hypoimmune iPSC-derived cardiomyocytes survive in immunocompetent allogeneic macaques [20]. Inhalable exosomes repair cardiac function in swine [21]. Cardiac organoids recapitulate myocardial infarction biology in a dish [39,43]. Volumetric bioprinting produces conduction-system-patterned cardiac tissue [27]. Single-nucleus transcriptomics has identified the CM4 regenerative cardiomyocyte subpopulation and its transcriptional regulators, and newer spatial work has identified a distinct, and previously overlooked, remote-endocardial injury-response pathway [22,23]. Deep learning begins to predict differentiation outcomes from tissue profiles [24,25]. Each of these advances addresses a specific barrier that has limited the field for years. Translating them from experimental demonstration to treatments that help patients with heart failure will require equal investment in clinical trial design, manufacturing standardisation, and regulatory clarity disciplines that are as essential to the field’s progress as any biological discovery, and that have not received equivalent attention.

## Figures and Tables

**Table 1:** **Cell-based and cell-free therapies for cardiovascular regeneration: cell sources, delivery routes, and key outcomes, in historical order of clinical introduction.**

Cell Origin/Era	Cell Type	Delivery Route (s)	Key Outcomes and Primary References	Reference
Skeletal muscle (1990s)	Autologous skeletal myoblasts	Intramyocardial	MAGIC trial halted: elevated ventricular arrhythmia incidence without durable benefit; lack of Cx43 prevents electrical integration	[28,29]
Bone marrow (early 2000s)	BM-MNCs, CD34+/CD133+ progenitors	Intracoronary, intramyocardial, intravenous	REPAIR-AMI: +2.5% LVEF at 4 months. In ACT34-CMI, intramyocardial autologous CD34+ cells reduced angina frequency and improved exercise tolerance in refractory angina. [16,30]	[15,16,30]
Bone marrow/umbilical cord/adipose (2000s–present)	MSCs, UC-MSCs, ADSCs	Intravenous, intramyocardial, transendocardial	POSEIDON confirmed acceptable safety of autologous and allogeneic MSC delivery without significant donor-specific alloimmune reactions. MSC-HF reduced LV end-systolic volume and improved LVEF at 12 months. [18,35]	[18,35]
Endogenous/biopsy-derived (2000s–2010s)	c-kit+ CPCs (SCIPIO, retracted); Cardiosphere-derived cells (CDCs)	Transendocardial, intracoronary, intrapericardial	CADUCEUS: scar mass 25.2 → 17.5% LV mass at 12 months. ALLSTAR: stopped for futility, infarct-size endpoint not met (*p* = 0.63 at 6 months, *p* = 0.39 at 12 months). Porcine dose-finding/intrapericardial data	[17,37,39]
Pluripotent stem cell-derived (2010s–present)	iPSC-CMs, hESC-CMs (±co-transplanted endothelial cells)	Epicardial patch/EHM, intramyocardial injection	Remuscularisation in primates and pigs; transient arrhythmias with immature grafts, resolving with maturation. Hypoimmune lines survive allogeneically in macaques without immunosuppression. Primate-and-human EHM allografts: long-term retention, structural remuscularisation	[20,43,47,49]
Cell-free (2020s, accelerating 2024–2025)	MSC-, PSC-, ADSC-derived exosomes; lung-spheroid-cell-derived exosomes (SCENT)	Intravenous, intracoronary, local hydrogel, inhalation	Anti-apoptotic, anti-fibrotic, angiogenic in preclinical models; inhaled SCENT improves cardiac function in mouse and swine via a pulmonary–cardiac anatomical axis and CD36-dependent mechanism; avoids immune rejection; enables outpatient repeated dosing	[21]

**Table 2:** **Cell-based versus cell-free (exosome/EV) cardiac regenerative platforms: a direct comparison.**

Attribute	Live-Cell Products (MSC, CDC, iPSC-CM, BM-MNC)	Exosome/EV Products (MSC-EV, Engineered Exosomes, SCENT)	Reference
Manufacturing cost & scalability	High: cell expansion, viability/potency QC, cold-chain (often cryopreserved) logistics; batch-to-batch variability from donor and passage number.	Potentially lower per-dose once a producer cell line is established, but no GMP-standardised isolation protocol yet exists (ultracentrifugation vs. size-exclusion vs. tangential-flow filtration have different yield/purity trade-offs).	[13,53]
Storage stability	Limited; cryopreservation causes viability/functional loss; most products require near-time-of-use preparation.	Exosomes are generally more freeze/thaw-stable than intact cells, but formal long-term stability and potency-retention data at clinical scale specific to cardiac exosome products are still limited.	[21]
In vivo half-life/dosing implication	Cells are largely cleared within 24 h regardless of type (<5% retained) [12,15]; single-dose paradigm dominates current trials.	Exosomes also have a short in vivo half-life, but their small size and lack of engraftment requirement make them compatible with repeated, low-burden dosing (e.g., inhaled SCENT was dosed on 7 consecutive days in preclinical models).	[21]
Feasible administration routes	Intracoronary, transendocardial, intramyocardial (surgical), intravenous (MSC only, low myocardial concentration).	All of the above, plus local hydrogel-sustained release and, uniquely, non-invasive inhalation via the pulmonary-cardiac circulation.	[21]
Immunogenicity/rejection risk	Allogeneic products require immune-privilege (MSC) or genetic engineering (hypoimmune iPSC) to avoid rejection; PSC-derivatives carry tumorigenicity risk from residual undifferentiated cells [20,31,54].	Exosomes lack surface MHC antigens and do not trigger cytotoxic immune responses at therapeutically relevant concentrations, sidestepping rejection biology entirely.	
Regulatory classification (current status)	Established biologic/cell-therapy pathway in principle; no FDA/EMA approval yet granted for MI or heart failure indications [55,56].	Unresolved in all major jurisdictions; whether a purified EV product is a biologic, a drug, or a combination product remains undetermined, which is actively slowing investment.	[57,58]
Human dosing evidence	Established across >200 registered trials; dose ranges and delivery volumes are relatively well characterised for BM-MNC/MSC/CDC products.	Not yet established in humans for any indication in cardiac repair; effective dose, dosing interval, and cargo-standardisation requirements remain open questions.	[51]

The purpose of this comparison is not to declare cell-free platforms categorically superior—human dosing and long-term safety data for exosome products are, if anything, less mature than for cell products—but to make explicit, attribute by attribute, why the mechanistic case (Section 2.2) points toward cell-free manufacturing as the more tractable long-term engineering problem, while acknowledging that regulatory and manufacturing standardisation for exosomes currently lags behind that of cell products.

**Table 3:** **Representative landmark preclinical and clinical studies evaluating cell-based therapies for cardiac regeneration.**

Trial	Phase/n	Disease	Cell Type/Delivery	Primary Endpoint	Outcome	References
REPAIR-AMI	Phase 2 RCT, n = 204	Acute MI	BM-MNCs, intracoronary	LVEF change at 4 months	+2.5% LVEF vs. control; not maintained at 5 years	[16]
BAMI	Phase 3 RCT, n = 375	Reperfused STEMI	Autologous BM-MNCs, intracoronary	All-cause mortality at 2 years	No mortality benefit (3.3% vs. 3.8%, *p* = 0.77); largest definitive negative BM-MNC trial	[60]
CADUCEUS	Phase 1 RCT, n = 25	MI, LVEF 25–45%	Autologous cardiosphere-derived cells, intracoronary	Safety at 6 months	Scar mass 25.2 → 17.5% LV mass; viable mass/contractility improved; LVEF change modest; sustained at 1 year	[17]
ALLSTAR	Phase 1/2 RCT, n = 142	Prior MI, LVEF <45%	Allogeneic CDCs (CAP-1002), intracoronary	% change in infarct size (MRI)	Stopped early for futility; primary endpoint not met (*p* = 0.63 at 6 months, *p* = 0.39 at 12 months); favourable secondary remodelling signals	[39]
POSEIDON	Phase 1/2 RCT, n = 30	Ischaemic cardiomyopathy	Autologous vs. allogeneic MSCs, transendocardial	30-day SAE rate	SAE 6.7%; no alloimmune reactions at 1 year; modest structural improvement	[18]
MSC-HF	Phase 2 RCT, n = 60	Ischaemic heart failure	Autologous bone-marrow-derived MSCs, intramyocardial	LV end-systolic volume	Reduced LVESV; improved LVEF; reduced scar mass at 12 months	[35]
DREAM-HF	Phase 3 RCT, n = 537	HFrEF	Allogeneic mesenchymal precursor cells (rexlemestrocel-L), transendocardial	Recurrent decompensated HF events	Primary NOT met (HR 1.17, 95% CI 0.81–1.69, *p* = 0.406); secondary MACE reduction in NYHA class II subgroup requires confirmatory trial	[14]
CardiAMP-HF	Phase 3, ongoing/allogeneic arm paused	Ischaemic HFrEF	Potency-screened autologous BM-MNCs (main arm); allogeneic MSC (complementary arm)	Composite functional/HF endpoint	Autologous arm ongoing; allogeneic arm enrolment paused 2023 pending DSMB review	[61,62]

**Table 4:** Key translational challenges, unresolved contradictions, and corresponding strategies under investigation.

Challenge	What Is Established	What Remains Unresolved	Strategy	References
Cell survival and retention	Over 95% of injected cells lost within 24 h; hostile ischaemic and inflammatory environment	Optimal preconditioning protocol not standardised across cell types; dose–response relationship in humans undefined	Metabolic preconditioning; hydrogel scaffold delivery; CXCR4 homing; repeated dosing protocols	[51,79]
Immune rejection	Hypoimmune iPSCs survive 4 months in immunocompetent allogeneic macaques without immunosuppression	Long-term safety in humans unknown; NK cell tolerance durability unconfirmed; regulatory pathway for hypoimmune products undefined	CRISPR HLA knockout with CD47/PD-L1; exosome-based cell-free delivery; local transient immunosuppression	[20,57,84]
Arrhythmic risk	iPSC-CMs cause transient arrhythmias in primates that resolve with graft maturation; no arrhythmias in early human case reports	Discrepancy between primate and human data unexplained; minimum safe maturation threshold not defined; monitoring duration in human cases may be insufficient	Multi-modal maturation protocols; scRNA-seq quality control against CM4 signature; large-animal pre-clinical testing; computational risk modelling	[22,43,45,46,50,55]
DREAM-HF endpoint disconnect	Phase 3 trial failed primary endpoint (HR 1.2, *p* = 0.406) in 537 patients	Secondary MACE benefit in NYHA II subgroup cannot be distinguished from chance positive finding without powered confirmatory trial	Powered confirmatory Phase 3 trial in NYHA Class II patients with MACE as primary endpoint	[14,63]
Manufacturing and scalability	GMP-grade cell production feasible; batch variability persistent; cryopreservation losses unresolved	Potency assay validated against clinical outcomes not achieved for any cell or exosome type; GMP protocols for exosome products not globally standardised	Automated bioreactors; scRNA-seq-based lot characterisation; standardised GMP protocols; exosome product classification resolution	[30,53,84]
Regulatory classification	No FDA or EMA approval for MI or HF; cell product BLA pathway established in principle	Regulatory classification of engineered exosome products (biologic, drug, or combination) unresolved in all major jurisdictions; hypoimmune product pathway undefined	Pre-IND regulatory engagement; ISSCR/FDA guidance updates; international harmonisation of GMP standards	[57,58]

**Table 5:** **Selected ongoing or recently paused trials of iPSC-CM and exosome-platform cardiac regenerative therapies.**

Trial/Product	Platform	Phase/Status	Population	Notes	Reference
CardiAMP-HF, autologous arm	Potency-screened autologous BM-MNCs, intramyocardial	Phase 3, ongoing	Ischaemic HFrEF	Cell selection by validated potency assay rather than unselected dose	[61]
CardiAMP-HF, allogeneic MSC arm	Allogeneic natural-killer-1R+ MSCs	Phase 3, enrolment paused (2023)	Ischaemic HFrEF, patients lacking favourable autologous cells	Paused following interim DSMB review; outcome not yet fully reported	[62]
Heartseed HS-001	iPSC-derived cardiomyocyte spheroids, surgical delivery with CABG	Early-phase, dosing initiated; low/high dose arms	Advanced heart failure	Primary endpoint: 26-week adverse-event incidence; secondary endpoints include LVEF and 6 min walk distance	[89]
EHM allograft programme (Zimmermann/Göttingen-DZHK, following [49])	Epicardial engineered heart muscle from iPSC-CMs + stromal cells	Early clinical evaluation following primate and single-human data	Advanced heart failure	First human case reported alongside the primate cohort; a formal clinical trial has been described as underway by the originating group	[49]
Allogeneic iPSC-CM patch (Osaka group, following porcine and case-series work)	hiPSC-CM epicardial sheet	Early-phase case series ongoing/expanding	Ischaemic cardiomyopathy	Builds on the porcine chronic-MI safety/efficacy data described in Section 2.1.5	[44]

## Data Availability

No new data were generated or analysed in this study. Data sharing is not applicable to this article.

## Abbreviations

AAV	Adeno-associated virus
BM-MNC	Bone marrow mononuclear cell
CAR	Chimeric antigen receptor
CDC	Cardiosphere-derived cell
CRISPR	Clustered regularly interspaced short palindromic repeats
CVD	Cardiovascular disease
dECM	Decellularised extracellular matrix
EHT	Engineered heart tissue
ESC	Embryonic stem cell
EV	Extracellular vesicle
GBD	Global burden of disease
GMP	Good manufacturing practice
hESC-CM	Human embryonic stem cell-derived cardiomyocyte
HLA	Human leukocyte antigen
iPSC	Induced pluripotent stem cell
iPSC-CM	Induced pluripotent stem cell-derived cardiomyocyte
LVEF	Left ventricular ejection fraction
MACE	Major adverse cardiovascular event
MHC	Major histocompatibility complex
MI	Myocardial infarction
MSC	Mesenchymal stromal cell
PLGA	Polylactic-co-glycolic acid
PSC	Pluripotent stem cell
UC-MSC	Umbilical cord-derived mesenchymal stromal cell
